# Tissue-growth-based synthetic tree generation and perfusion simulation

**DOI:** 10.1007/s10237-023-01703-8

**Published:** 2023-03-04

**Authors:** Hyun Jin Kim, Hans Christian Rundfeldt, Inpyo Lee, Seungmin Lee

**Affiliations:** 1 Mechanical Engineering, Korea Advanced Institute of Science and Technology, 291 Daehak-ro, Yuseong-gu, Daejeon, 34141 Republic of Korea; 2 Mechanical Engineering, Kalsruhe Institute of Technology, Kaiserstraße 12, Karlsruhe, 76131 Germany

**Keywords:** Blood flow simulation, Growth-based tree generation, Multiscale modeling, Perfusion simulation

## Abstract

Biological tissues receive oxygen and nutrients from blood vessels by developing an indispensable supply and demand relationship with the blood vessels. We implemented a synthetic tree generation algorithm by considering the interactions between the tissues and blood vessels. We first segment major arteries using medical image data and synthetic trees are generated originating from these segmented arteries. They grow into extensive networks of small vessels to fill the supplied tissues and satisfy the metabolic demand of them. Further, the algorithm is optimized to be executed in parallel without affecting the generated tree volumes. The generated vascular trees are used to simulate blood perfusion in the tissues by performing multiscale blood flow simulations. One-dimensional blood flow equations were used to solve for blood flow and pressure in the generated vascular trees and Darcy flow equations were solved for blood perfusion in the tissues using a porous model assumption. Both equations are coupled at terminal segments explicitly. The proposed methods were applied to idealized models with different tree resolutions and metabolic demands for validation. The methods demonstrated that realistic synthetic trees were generated with significantly less computational expense compared to that of a constrained constructive optimization method. The methods were then applied to cerebrovascular arteries supplying a human brain and coronary arteries supplying the left and right ventricles to demonstrate the capabilities of the proposed methods. The proposed methods can be utilized to quantify tissue perfusion and predict areas prone to ischemia in patient-specific geometries.

## Introduction

Technologies associated with image-based blood flow simulations have evolved significantly over the last decades and some of the technologies are starting to be adapted in clinical applications (Colombo et al. 2022; Gutiérrez et al. 2021; Khan et al. 2021; Marsden 2014; Nørgaard et al. 2014; Plitman Mayo et al. 2020; Regazzoni et al. 2022; Schiavazzi et al. 2017; Schollenberger et al. 2021; Taylor and Figueroa 2009). The step towards actual clinical applications, however, is still challenging as there is much uncertainty in modeling patient-specific blood flow and pressure using medical image based geometries. Some of the challenges include modeling the physiology and pathophysiology of the vascular system beyond the image data resolution, estimating the dynamics of the system due to various autoregulatory mechanisms and modeling the interactions between blood motion and complex biological tissues. Especially, as realistic blood flow simulations require knowledge in the domains unseen in the medical image data, estimating patient-specific boundary conditions is a big challenge in adapting these technologies to actual clinical applications.

In recent years, several studies investigated relationships between major arteries that can be segmented using input medical image data and tissues where these vessels bifurcate and supply blood to Ide et al. (2017), Kaimovitz et al. (2005), Kang et al. (2019), Kim et al. (2016), Kurata et al. (2015), Malkasian et al. (2018), Sinclair et al. (2015), van den Wijngaard et al. (2013) and Van Horssen et al. (2016). Many of the studies rely on the physiological principle that the vessels supply blood to nearby tissues to minimize energy expenditure. Several computational methods such as Voronoi parcellation, minimum cost path, and scaling law based tessellation strategies have been proposed and compared against measurement data (Ide et al. 2017; Kang et al. 2019; Kim et al. 2016; Kurata et al. 2015; Malkasian et al. 2018; van den Wijngaard et al. 2013; Van Horssen et al. 2016). These studies provide a mapping between arteries and perfusion territories of the tissue and enable a patient-specific modeling of boundary conditions which are pre-requisite for blood flow simulations. Further, by means of comparison to experimental and empirical results, these studies have demonstrated key physiological principles such as blood vessels supplying nearby tissues and allometric scaling laws (West et al. 1997).

Additionally, vascular networks beyond the image data resolution can be constructed and used for simulated blood perfusion studies. Synthetic tree generation relies on the principle that the blood vessels supply the tissues by filling the tissue volume while minimizing required work. Previous tree generation strategies include constrained constructive optimization, staged growth-based constrained constructive optimization, nonlinear programming based constrained constructive optimization, and supply–demand relationship based tree generation methods (Blanco et al. 2021; Capasso et al. 2013; Coppini et al. 1997; Heck et al. 2015; Jaquet et al. 2019; Jessen et al. 2022; Karch et al. 1999, 2000; Levine et al. 2001; Milde et al. 2013; Mittal et al. 2005; Perfahl et al. 2017; Shen et al. 2021; Schreiner et al. 2006; Smith et al. 2000; Spill et al. 2015; Tawhai et al. 2000; Talou et al. 2021; Wang and Bassingthwaighte 1990; Yang and Wang 2013). The constrained constructive optimization (CCO) methods are the most widely used tree generation algorithms so far (Blanco et al. 2021; Jaquet et al. 2019; Karch et al. 1999, 2000; Schreiner et al. 2006; Talou et al. 2021). They rely on the principle that the synthetic trees are constructed by minimizing the energy required to maintain the blood vessel networks. This algorithm, however, has limitations as it seeks for a global optimization and the parallelization of the algorithm requires additional assumptions. Furthermore, when the algorithm is implemented for multiple sources, they compete with each other while filling the tissues and may require additional constraints to develop more realistic vascular networks (Jaquet et al. 2019; Papamanolis et al. 2021). Recently, there was a study to optimize the algorithm by executing with a domain decomposition strategy and this approach improved the execution time (Blanco et al. 2021). The staged growth-based constrained constructive optimization methods were developed to fill the prescribed biological tissues based on the observation that some blood vessels fill certain areas of the tissues first before supplying the remaining areas of the tissue domain (Karch et al. 2000; Talou et al. 2021). This approach has been implemented for various geometries and represented the anatomy observed in the cardiovascular system well. However, the tissue domain needs to be predefined before building synthetic trees. The nonlinear programming based constrained constructive optimization was recently developed to optimize the tree generation while searching over a larger space of candidate tree configurations and it was shown to further minimize the metabolic work of the synthetically generated trees compared to that of the constrained constructive optimization (Jessen et al. 2022). However, as the algorithms seek for a global minimum the execution may take longer than that of the constrained constructive optimization algorithms and heuristic assumptions can be implemented to reduce computational expense. The supply–demand based tree generation methods were implemented for idealized and actual patient-specific models but the methods were utilized with a simplification which omits the optimization that minimizes the work required to maintain the vascular system (Di Gregorio et al. 2021; Tawhai et al. 2000; Wang and Bassingthwaighte 1990; Yang and Wang 2013). As the generated vascular trees were not compared against the trees generated with the minimum work principle, the supply–demand based methods need to be further validated to see whether the generated tree structures support the minimum work principle.

The synthetic tree generation methods can be combined with perfusion simulation frameworks to estimate blood perfusion through the tissues and to predict the risk of tissue ischemia when there is an occlusion in a major artery. Previous perfusion simulations were conducted using tree networks generated with either experimental data or aforementioned tree generation algorithms. Smith et al. and Hyde et al. proposed a multiscale perfusion simulation framework by coupling the left myocardium with one-dimensional vascular trees which were obtained from animal models (Hyde et al. 2014; Smith et al. 2000). Lee et al. implemented a perfusion framework for idealized and porcine coronary data by coupling the perfusion solver with the ventricle mechanics (Lee et al. 2015). Michler et al. proposed a multi-compartment porous model in a porcine myocardial tissue using a homogenization method whereby multiscale vessel models are approximated to compartment models (Michler et al. 2013). Most of the works have been implemented for ideal geometries and have not utilized patient-specific geometries and parameters for the tree growth and perfusion simulations. Recently, Papamanolis et al. implemented a perfusion simulation framework for patient-specific coronary arteries (Papamanolis et al. 2021) and Di Gregorio et al. implemented a perfusion framework for patient-specific coronary arteries with different length scales of the vessels using a multi-compartment porous medium model (Di Gregorio et al. 2021).

The main purpose of this paper is to develop a synthetic tree generation algorithm which constructs anatomically realistic vessel networks that observe physiological principles. Further, short execution time is required for the algorithm to be utilized effectively. To achieve this, we develop a tree generation algorithm which functions based on tissue growth and succeeding vascular growth. It ensures that blood vessels supply nearby tissues and vascular trees are generated to meet the metabolic demand of the supplied tissues while obeying the principle of minimum work. The proposed algorithm is parallelized to minimize computational expense. Finally, the synthetically generated vessels are combined with a perfusion simulation framework. Tree generation and perfusion simulation are demonstrated for an idealized geometry with different tree resolutions and metabolic demands for validation as well as two patient-specific geometries as representative examples.

## Methods

This section is organized in three subsections. The first section describes key principles and implementation of the algorithm proposed to generate synthetic vascular networks. Next, the governing equations for simulating blood flow and pressure in the generated vascular network models and a Darcy flow model are explained. Last section briefly introduces the steps of constructing patient-specific geometries from medical image data.

### Tissue Growth-based Synthetic Tree Generation

The vascular networks consist of two domains. The first domain is constructed from medical image data by segmenting major arteries which are visible within the input image resolution. The construction process is explained in the third subsection. The other domain consists of artificially generated networks of trees representing the vessels that are below the image resolution in vessel size. The construction process of these synthetic trees is explained in this subsection.

**Fig. 1:**
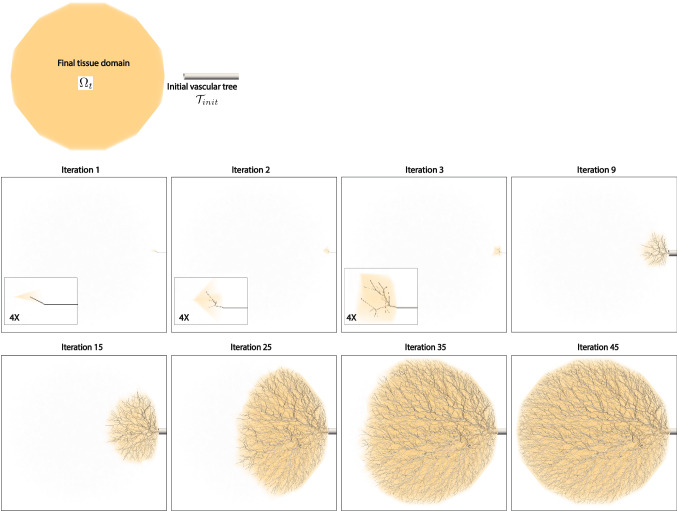
Captured pictures while generating vascular trees using the growth-based optimization algorithm. The initial vascular tree starts with a user-defined minimum radius and the tissue that satisfies the metabolic demand with the vessel. As the tissue grows to neighboring tissues the vessels grow accordingly to satisfy the increased metabolic demand. The radius at the source location grows up to the original radius value of the initial vascular tree. Note that the grown tissues and generated vessels are scaled by four folds at the bottom left boxes for the captured images at iteration 1, 2, and 3

First, the initial vascular tree ${\mathcal {T}}_{{init}}$ is defined. ${\mathcal {T}}_{{init}}$ consists of major arteries which are segmented from the input image data. All outlets of the segmented vessels are potential source locations to grow synthetic vessels from. If the segmented outlets are located inside the tissue or within a set distance criteria from the tissue, they are defined as eligible source locations. The source locations are assumed to be the initial locations that initiate the growth of the tissues and the blood vessels from. Once these source locations are identified, the radius values are scaled down to a user-defined minimum vessel radius $r_{min}$ as shown in Fig. 1. Flow supplied by the initial source locations is estimated using Murray’s law or power law where $q_{i} \propto {r}_{min}^{k}$ for each source location *i*. Here, $q_{i}$ refers to the flow rate supplied by the *i*th source location. The power law exponent *k* can be selected depending on the characteristics of the vessel network. The initial tissue volume supplied with this flow is determined using the allometric scaling laws whereby flow is proportional to the tissue volume to the *p*th power. *p* is an exponent determined based on empirical and analytic studies (West et al. 1997). The location of the initial tissue $\Omega _{{init}}$ is determined by claiming the volume starting from the tissue closest to the vascular outlet until the required blood flow of the volume with its specific metabolic demand matches the outlet flow of the vascular segment.

Once the initial source locations and initial tissues are defined, the initial tissues grow and trigger the creation of new vessels and growth of existing vascular trees. The tissue starts from the initial tissue $\Omega _{{init}}$ perfused by the initial tree ${\mathcal {T}}_{{init}}$ and grows toward a final tissue $\Omega _{t}$ which is segmented from the medical image data. The tissue growth rate can be either isotropic or anisotropic. The growth rate and direction are determined by the user-defined growth rate vector ${\textbf {c}}_{i,p}$ for each source location ${\textbf {x}}_{i,p}$ at *i*th iteration. Tissues and vascular vessels grow in tandem, so the tissue growth occurs first near the vascular networks and the corresponding vessels grow subsequently. Each tissue partition perfused by a terminal segment grows independently until the tissue growth is completed by growing into the final tissue $\Omega _{t}$.

For a given tissue $\Omega _{i}$ perfused by a vascular tree ${\mathcal {T}}_{i}$ at *i*th iteration, the following Voronoi diagram is computed for each partition of the tissue $\Omega _{i,p}$ perfused by an individual terminal segment with a terminal location ${\textbf {x}}_{i,p}$ for a given growth rate vector ${\textbf {c}}_{i,p}$:

1 $$\begin{aligned}&{\mathcal {R}}_{i,p}({\mathcal {T}}_{i}, {\Omega }_{i,p}) = \{{\textbf {x}} \in \Omega _{t}, \, {\textbf {x}} \not \in \Omega _{i,k} \text { for } k = 1, \dots , n_{i} \nonumber \\&{\Vert \, \Vert {\textbf {c}}_{i,p,n} \cdot ({\textbf {x}}_{n} - {\textbf {x}}_{i,p,n}) \Vert < min \Vert 1, \Vert {\textbf {c}}_{i,k,n} \cdot ({\textbf {x}}_{n} - {\textbf {x}}_{i,k,n}) \Vert \Vert ,} \nonumber \\&\quad \text {where } k \ne p, \text { for } n = 1, \dots , n_{sd} \} , \end{aligned}$$

where ${\mathcal {R}}_{i,p}({\mathcal {T}}_{i}, {\Omega }_{i,p})$ is the tissue partition at *ith* iteration perfused by an individual terminal segment located at ${\textbf {x}}_{i,p}$, $n_{sd}$ is a spatial dimension of the tissues and the vascular networks, and $n_{i}$ is the number of terminal segments at the *i*th iteration.

Tissues at the ${(i+1)}$th iteration are constructed by a union of ${\mathcal {R}}_{i,p}({\mathcal {T}}_{i}, {\Omega }_{i,p})$ for each terminal segment with $\Omega _{i}$, the tissue at *i*th iteration, where $\Omega _{i+1} = \Omega _{i} \cup {\mathcal {R}}_{1}({\mathcal {T}}_{i}, {\Omega }_{i,1}) \cup \dots \cup {\mathcal {R}}_{n_{i}}({\mathcal {T}}_{i}, {\Omega }_{i,n_{i}})$.

After the tissue grows to $\Omega _{i+1}$ the vessels grow in tandem to satisfy the metabolic demand of the newly grown tissue. The number of new terminal segments $n_{i+1}$ is obtained by computing the metabolic demand of the newly grown tissue $\Omega _{i+1}$:

2 $$\begin{aligned} {Q}_{\Omega _{i+1}} = \sum _{p=1}^{n_{t,i+1}} m_{t,p} v_{p}, \end{aligned}$$

where $n_{t,i+1}$ is the number of tissue partitions in $\Omega _{i+1}$, $m_{t,p}$, the metabolic demand of each tissue partition $t_{p}$ and $v_{p}$ is the volume of the tissue partition $t_{p}$. The metabolic demand of a single tissue partition is assumed to have a constant value.

The flow increment, ${Q}_{\Omega _{i+1}} - {Q}_{\Omega _{i}}$ determines the number of terminal segments to be generated. The minimum flow $q_{min}$ of a single terminal segment is set based on the pre-defined minimum radius $r_{min}$. The number of terminal segments $n_{i+1}$ is determined using the following expression:

3 $$\begin{aligned} n_{i+1} = {round}\left( \frac{{Q}_{\Omega _{i+1}} - {Q}_{\Omega _{i}}}{q_{min}}\right) . \end{aligned}$$

New terminal segments are generated and connected to the existing vascular trees to supply blood to the newly grown tissue $\Omega _{i+1}$. We define the new merged vascular trees as ${\mathcal {T}}_{i+1}$. The new vascular trees ${\mathcal {T}}_{i+1}$ are generated to minimize work required to supply blood to the grown tissue $\Omega _{i+1}$ and maintain the vascular trees ${\mathcal {T}}_{i+1}$. The loss function ${\mathcal {L}}({\mathcal {T}}_{i+1}, {Q}_{\Omega _{i+1}})$ is defined as follows:

4 $$\begin{aligned} {\mathcal {L}}({\mathcal {T}}_{i+1}, {Q}_{\Omega _{i+1}}) = \sum _{p=1}^{n_{tot,i+1}}\left\{ \frac{8 \mu {l}_{p}}{\pi {r}_{p}^{4}}q_{p}^{2} + {\beta }_{p}\pi {r}_{p}^2 {l}_{p}\right\} . \end{aligned}$$

The first term refers to viscous work due to blood flow and the latter approximates the metabolic work of the vascular networks. Here, ${r}_{p}$, $l_{p}$, and $q_{p}$ are the radius, length, and flow of a synthetically generated segment *p* respectively. $\mu$ is the viscosity of blood and ${\beta }_{p}$ is a metabolic work coefficient for the synthetic segment *p*. Further, $n_{{tot},i+1}$ is the number of all synthetically generated segments. All the required parameters are defined in the optimization parameter set ${\mathcal {P}}_{{opt}}$.

When generating vascular trees, we assume that they have a binary bifurcation structure and satisfy pre-defined geometric constraints. The power law ${r}_{0}^{k} = {r}_{1}^{k} + {r}_{2}^{k}$ is obeyed for each bifurcation where *k* is a power law exponent, ${r}_{0}$ is the radius of a parent vessel, and ${r}_{1}$ and ${r}_{2}$ are the radii of the daughter vessels. All the required parameters including the power law exponent *k* are defined in the geometric parameter set ${\mathcal {P}}_{{geo}}$. Additionally, based on a supply and demand relationship between the vascular trees and perfused tissues, new vascular trees ${\mathcal {T}}_{i+1}$ adapt in size according to the metabolic demand of the perfused tissues. The relationship obeys a power law such that the flow rate $q_{p}$ for a segment with the radius $r_{p}$ can be described with the following relationship: $q_{p} \propto {r}_{p}^{k}$ and $q_{p} \propto {\mathcal {R}}_{i,p}({\mathcal {T}}_{i}, {\Omega }_{i,p})$.

The initial tree starts from a radius $r_{{min}}$ in vessel size at each initial source location. As the tissues and trees grow, the diameter at the initial source location increases accordingly. The tree generation from the initial source location is completed when the radius at the source location is equal to or exceeds the actual segmented source radius, which is obtained from the segmentation of input medical image data. The same principle is obeyed when applying the tree generation algorithms to multiple sources. Additionally, when multiple sources compete to fill the given tissue, the growth rate is monitored for each source to ensure that these sources grow with a uniform growth rate. As the trees grow and supply tissue volumes, source locations with larger diameters will eventually require more iterations to grow fully as they supply more flow. The perfusion territories will be affected depending on how the growth rates are prescribed for multiple sources. For this study, we assumed that each source has the same growth rate for simplicity.

Using these principles, synthetic vascular trees are constructed to obey the supply and demand relationship between blood vessels and supplied tissues. As the perfused tissues grow in size, the metabolic demand of the tissues increases and the vascular trees that supply blood to them grow accordingly to satisfy the increased demand while obeying the provided physiological principles.
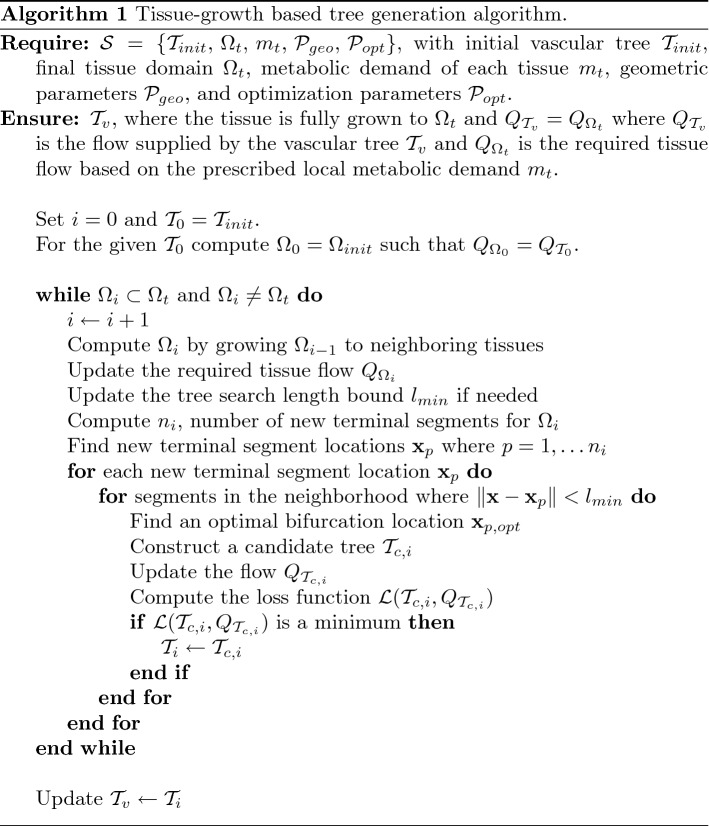


Here, the tree search length ${l}_{{min}}$ constrains the search domain when searching for candidate locations to generate vessels from. The terminal segment locations ${\textbf {x}}_{p}$ can be determined using multiple ways. For this study, the centroid locations of the discretized tissue volume mesh are used as the terminal segment locations but they can be determined independent of the tissue volume mesh.

Further, a constrained constructive optimization algorithm was implemented to compare against the proposed tissue-growth based tree generation algorithms. For the details on the algorithm, refer to the articles by Jaquet et al. (2019), Karch et al. (1999) and Talou et al. (2021).

### Governing blood flow equations

This subsection consists of three parts: one-dimensional blood flow equations to solve for blood flow and pressure in the vascular networks, a three-dimensional perfusion model to solve for blood flow and pressure occurring in the small arteriole and capillary beds of the tissues, and the coupling of the one-dimensional blood flow equations and the perfusion model.

#### Blood flow in networks of vessels

For the generated vascular networks, blood flow is modeled with a one-dimensional approximation of the Navier–Stokes equations. The deformability of the blood vessel walls was ignored for this study. The mass conservation and the linear momentum balance equations for each vessel segment in the flow direction *z* are solved as follows:

5a $$\begin{aligned}{} & {} \frac{\partial Q}{\partial z} = 0 \end{aligned}$$

5b $$\begin{aligned}{} & {} \frac{\partial Q}{\partial t} + \frac{\partial }{\partial z} \left[ (1 + \delta ) \frac{Q^{2}}{S}\right] + \frac{S}{\rho } \frac{\partial p}{\partial z} = Sf + N \frac{Q}{S}, \end{aligned}$$

where *Q* is the flow rate, *p* is the pressure, and *S* is the cross-sectional area of the vessel along the *z* coordinate. *f* is an external force, $\delta$ is a profile function related parameter, *N* is the viscosity related parameter and $\nu$ is the kinematic viscosity. Note that for a parabolic velocity profile function, $\delta =\frac{1}{3}$ and $N=8 \pi \nu$.

These equations are solved for networks of vessels by applying conservation of mass and continuity of pressure at each junction. At the inlets of the vascular trees, an inflow is assigned as a boundary condition and at each terminal vessel of the networks, a resistance or three-element Windkessel model is assigned as a boundary condition.

The system of equations is solved in an iterative fashion for a given time step until flow rate difference between two consecutive iterations decreases below a pre-defined threshold. A generalized-$\alpha$ method is utilized for the time integration (Hughes and Lubliner 1973; Jansen et al. 2000; Papamanolis et al. 2021).

#### Blood flow in tissues

Blood flow perfused in the tissue is simulated using Darcy’s law. For the tissue domain given by $\Omega$ with the boundary $\Gamma$, Darcy’s law for the flow of a viscous fluid in a permeable medium and conservation of mass are given as follows:

6a $$\begin{aligned}{} & {} \vec {v} = -\frac{\kappa }{\mu } \left( \nabla p + \frac{\rho }{g_{c}} \vec {g}\right) \text { in } \Omega \end{aligned}$$

6b $$\begin{aligned}{} & {} \nabla \cdot \vec {v} = \beta _{\text {source}} \left( p_{\text {source}} - p\right) - \beta _{\text {sink}} \left( p - p_{\text {sink}}\right) \text { on } \Omega \end{aligned}$$

6c $$\begin{aligned}{} & {} \vec {v} \cdot \vec {n} = \psi \text { on } \Gamma , \end{aligned}$$

where $\vec {v}$ is the Darcy velocity vector, *p* is the capillary pressure, $\vec {g}$ is the gravity vector, $\mu$ is the blood viscosity, $\kappa$ is the permeability, $\rho$ is the blood density, ${g}_{c}$ is the conversion constant, $\psi$ is the normal component of the velocity assigned on the boundary, and $\vec {n}$ is the unit outward normal vector at the boundary $\Gamma$. $p_{\text {source}}$ and $p_{\text {sink}}$ are the source and sink pressure terms respectively and $\beta _{\text {source}}$ and $\beta _{\text {sink}}$ are pressure-coupling coefficients which represent the conductance of flow entering and exiting the tissues, respectively.

Sources and sinks represent the flow entering the tissue through the synthetic terminal vessels and the drainage through the venous system. Zero flow boundary condition with $\psi =0$ is assigned on the boundary $\Gamma$ of the tissues (Papamanolis et al. 2021). A stabilized mixed Galerkin finite element method is implemented with linear shape functions for both velocities and pressure (Masud and Hughes 2002). A generalized-$\alpha$ method is used for the time integration scheme (Jansen et al. 2000).

For the following perfusion simulations, the pressure-coupling coefficients are initialized as follows:

7a $$\begin{aligned}{} & {} {\beta _{\text {source}} = \frac{{\bar{m}}_{t}}{{\bar{p}}_{\text {source}} - {\bar{p}}}} \end{aligned}$$

7b $$\begin{aligned}{} & {} {\beta _{\text {sink}} = \frac{{\bar{m}}_{t}}{{\bar{p}} - {\bar{p}}_{\text {sink}}}} \end{aligned}$$

where ${\bar{m}}_{t}$ is the average metabolic demand of the tissue domain $\Omega$, ${\bar{p}}_{\text {source}}$ is the average source pressure, ${\bar{p}}_{\text {sink}}$ is the average sink pressure, and ${\bar{p}}$ is the average capillary pressure.

#### Explicit coupling between vessel networks and tissues

**Fig. 2:**
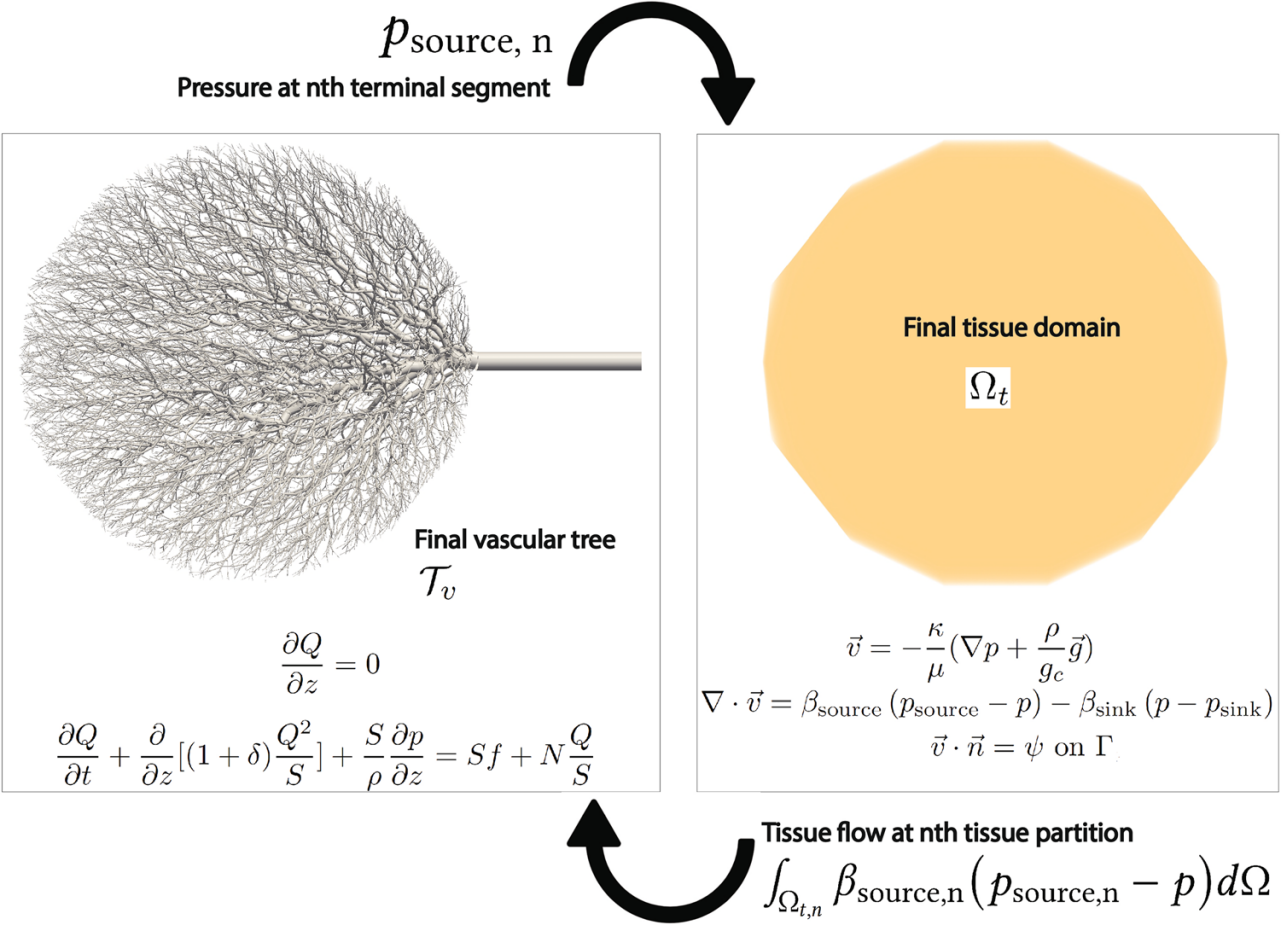
Explicit coupling between vessel networks and tissues happens by passing computed terminal pressure values from the one-dimensional blood flow solver to the perfusion solver and passing the tissue flow of each tissue element from the perfusion solver back to the one-dimensional blood flow solver

The synthetic vascular trees transport blood to the tissues and blood perfusion in the tissues occurs through small arteriole and capillary vessels. Blood flow and pressure of the synthetic vascular trees are solved with one-dimensional blood flow equations. Blood flow and pressure in the capillaries in the tissues are computed using Darcy’s law. The coupling of the two equations is done explicitly by feeding the computed pressure of the terminal segments as source pressures of Darcy’s flow equations as demonstrated in Fig. 2. The computed flow using Darcy’s flow equations is fed back to the one-dimensional blood flow equations. The explicit coupling is conducted iteratively within the same time step until both the pressure and flow of terminal segments converge within pre-defined criteria. The pressure-coupling coefficient values are adjusted slightly from the initially set values to match the pressure and flow values of terminal segments if needed.

### Construction of patient-specific geometries using medical image data

Patient-specific geometries utilized in the results session were constructed using ITK-SNAP, Slicer, Autodesk MeshMixer (https://www.meshmixer.com/), Openflipper, and SimVascular (Fedorov et al. 2012; Kauke et al. 2019; Möbius and Kobbelt 2012; Updegrove et al. 2016). The lumen boundary of the blood vessels was segmented using a level set and threshold method of ITK-SNAP and was post-processed using SimVascular and Openflipper to generate both three-dimensional and one-dimensional discretized models. The tissue models were generated using the same tools. Slicer was used additionally to manually correct the tissue models as the rule-based approaches sometimes failed. Autodesk MeshMixer was used to smooth the generated surface meshes.

## Results

In this section, we generate artificial vascular networks for ideal geometries as well as two patient-specific geometries to validate the implementation of the proposed methods and to demonstrate the capabilities of them. An ideal geometry is chosen to show that artificial networks of vessels of different density can be generated while obeying the supply-and-demand relationship between the vessels and the tissues. Additionally, the metabolic demand of the tissues were modified to show that this supply-and-demand relationship is preserved for more general cases. Lastly, the outputs of the proposed methods were compared against that of the constrained constructive optimization methods.

For the two patient-specific geometries, cerebrovascular arteries with the left and right hemispheres and coronary arteries with the left and right ventricles were chosen. The left and right brain hemispheres were assigned a uniform metabolic demand value, whereas for the left and right ventricles a different metabolic demand value was assigned.

### Synthetic Tree Generation and Perfusion Simulation of an ideal geometry

In this subsection, a spherical model with a center located at (0, 0, 0) and a radius of 10 cm was used as an input tissue to be perfused. The synthetic vascular tree networks were generated from two source locations which are located at (0, 0, $-10$) and (0, 0, 10). Both root vessels have an identical radius value of 0.4 cm. The synthetic trees were generated for tissues with prescribed metabolic demand values for different numbers of terminal segments. Blood flow and pressure of the generated vessels and perfusion in the tissues were computed using the proposed perfusion simulation framework.

#### Synthetic Tree Generation and Perfusion Simulation with different tree resolutions

The synthetic vessels were generated with different tree resolutions. The number of generated synthetic terminal segments varies between 567, 1189, 2369, 5003, 11,192, and 24,493. The metabolic demand of the tissues is uniform at 1.0 ml/min/g and the tissue density is uniform at 1.05 g/cm$^{3}$. The perfusion simulation is conducted assuming a uniform permeability coefficient of 0.000107/mmHg/s. The sink pressure is set at 0 mmHg and the pressure-coupling coefficients $\beta _{{source}}$ and $\beta _{{sink}}$ are set at 0.000281 and 0.00117/mmHg/s, respectively.

**Table 1:** Tissue volume, flow, and vascular volume data of the generated synthetic trees with different tree resolutions

Number of terminal segments	567	1189	2369	5003	11,192	24,493
Tissue volume supplied by the left source (ml)	1765.8	1695.6	1770.8	1803.9	1771.4	1796.9
Tissue volume supplied by the right source (ml)	1815.3	1888.9	1813.1	1779.9	1813.2	1787.9
Ratio	0.97	0.90	0.98	1.01	0.98	1.01
Total flow of the left source (ml/s)	30.9	29.7	31.0	31.6	31.0	31.4
Total flow of the right source (ml/s)	31.8	33.0	31.7	31.1	31.7	31.3
Ratio	0.97	0.90	0.98	1.02	0.98	1.00
Tree volume of the left source (ml)	11.82	12.29	13.07	14.33	15.98	17.06
Tree volume of the right source (ml)	11.95	12.96	13.27	14.46	15.70	17.05
Ratio	0.99	0.95	0.98	0.99	1.02	1.00

The perfusion simulations were conducted for steady flows for this study. For the one-dimensional blood flow equations, the flow at each terminal segment was determined by the metabolic demand of the perfused tissue. The perfusion volume and flow of each source for different numbers of terminal segments are presented in Table 1. Both source locations have similar perfusion volumes and flows as they have the same source radius values. The synthetic tree volume increases as the number of terminal segments increases as it continues bifurcation to smaller vessels. Note that the tissue geometry was generated using a coarse edge length and the total tissue volume is 3581.1 ml.

**Fig. 3:**
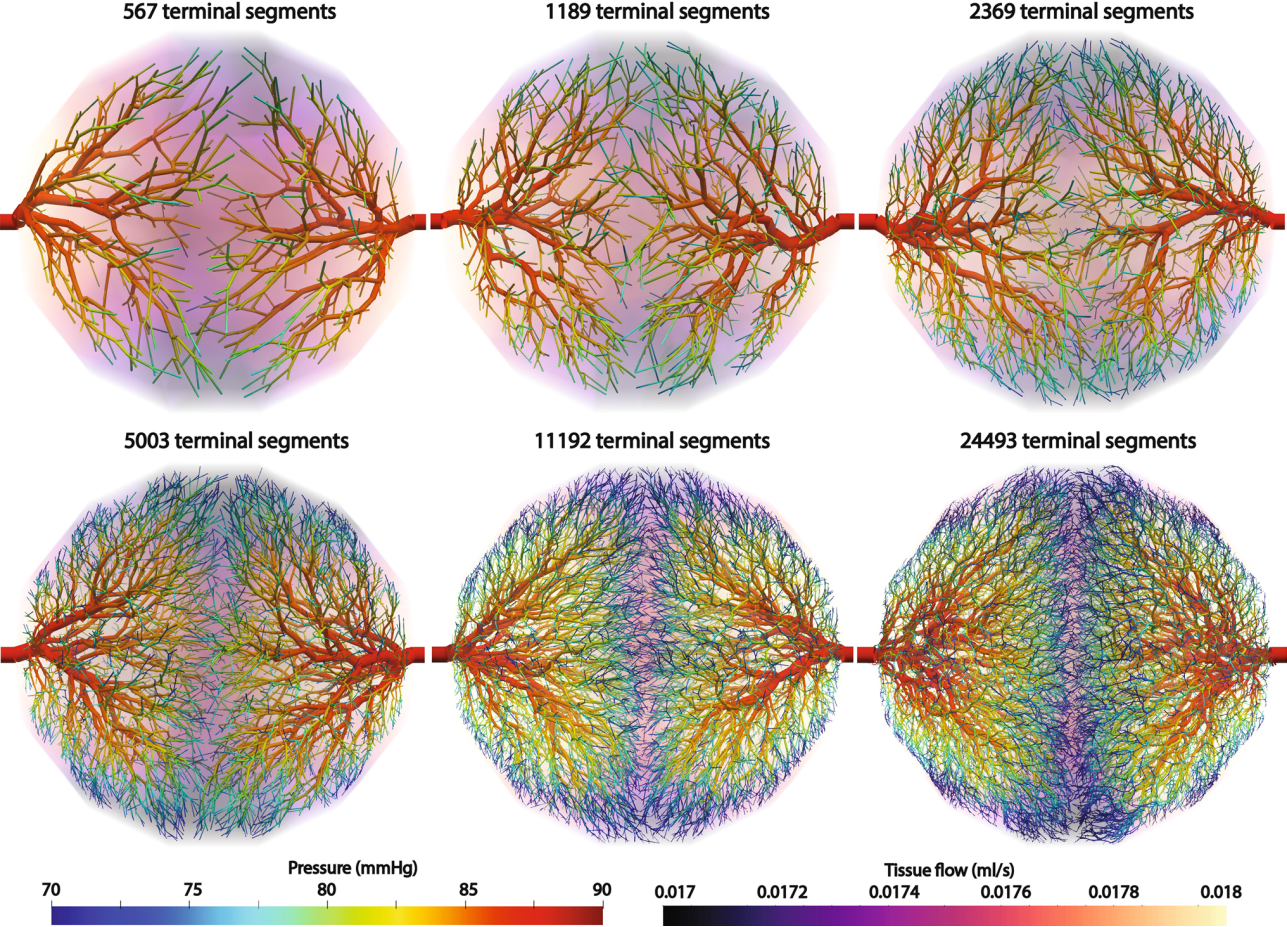
Generated synthetic tree networks and simulated perfusion with different number of terminal segments. Pressure distribution of the synthetic trees is displayed on the surface of the synthetic trees whereas the tissue flow per each volume mesh element is plotted for the tissue domain

Figure 3 displays the generated synthetic trees, the pressure distribution in the vessels, and the computed blood perfusion on the tissues. The generated synthetic trees and the perfused volumes are symmetric as both sources have identical source radius values and have the same growth rate when constructing synthetic trees. The tissue flow supplied by the synthetic trees is uniform with small reductions distant from the source locations. These reductions are caused by pressure losses as the blood travels toward the terminal segments.

#### Synthetic tree generation and perfusion simulation with non-uniform metabolic demand of the tissue

The synthetic vessels were generated for the same tissue volume with variable metabolic demands. In the first considered case, the metabolic demand of the tissue decreases radially from 3.0 ml/min/g at the center to 0.3 ml/min/g in the periphery. For the second simulated case, the metabolic demand increases radially from 0.1 ml/min/g at the center to 1.5 ml/min/g in the periphery. Note that the maximum and minimum metabolic demand values are slightly modified to preserve the same total required flow for the tissue. The perfusion simulation was conducted assuming the permeability coefficient is 0.000107/mmHg/s. The sink pressure is set at 0 mmHg and the pressure-coupling coefficients $\beta _{source}$ and $\beta _{sink}$ are initially set at 0.000281/mmHg/s and 0.00117/mmHg/s, respectively. The pressure-coupling coefficient $\beta _{{sink}}$ was adjusted slightly within 2.0 % to match the prescribed total flow in the tissue.

**Fig. 4:**
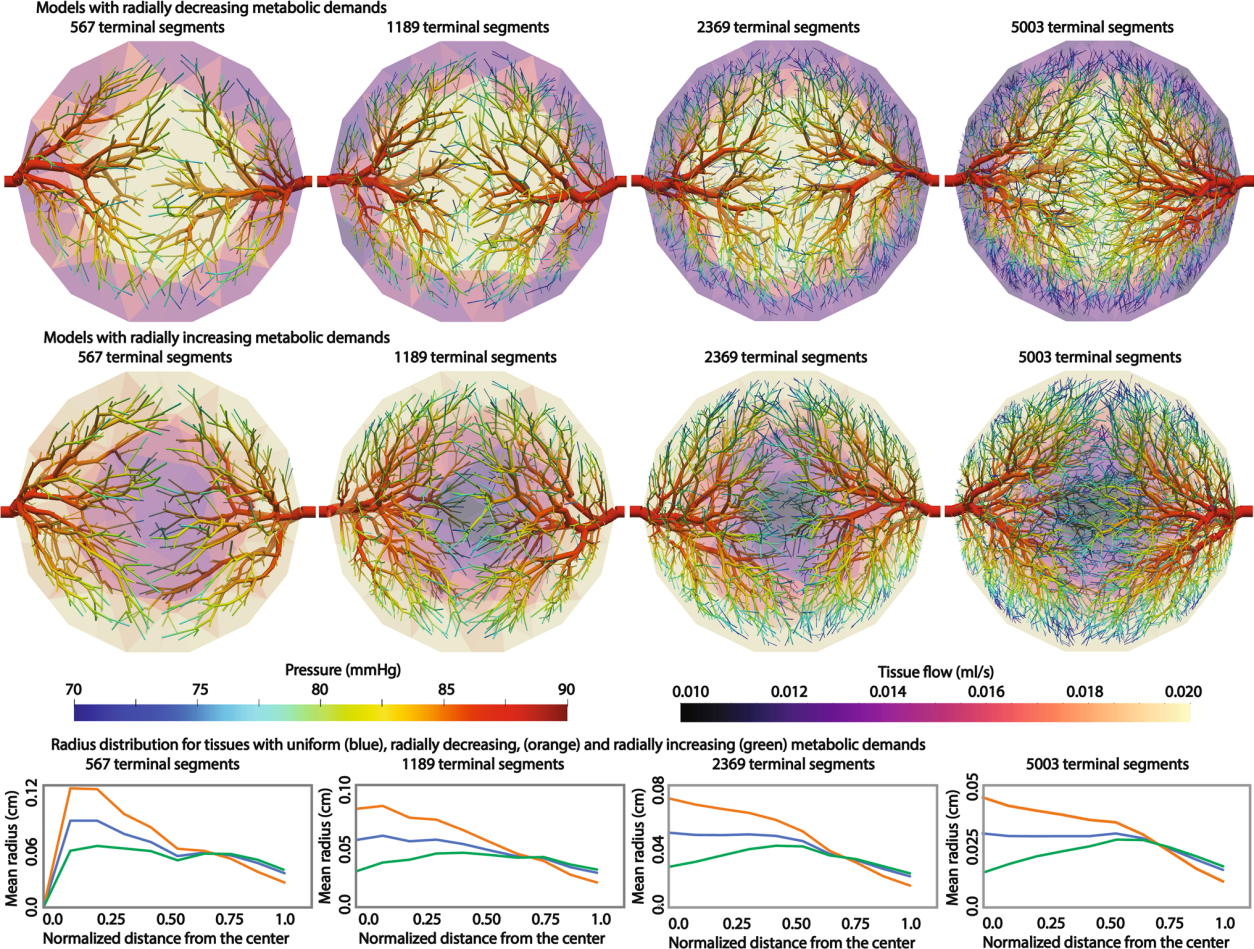
Generated synthetic tree networks and simulated perfusion with different number of terminal segments with radially decreasing metabolic demand (top), radially increasing metabolic demand (middle), and the terminal radius distribution as a function of normalized distance from the center for the generated synthetic trees with uniform, radially decreasing, and radially increasing metabolic demands (bottom). Pressure distribution of the synthetic trees is displayed on the surface of the synthetic trees whereas the tissue flow per each volume mesh element is plotted for the tissue domain. The radius distribution of uniform metabolic demand is plotted in blue whereas the distribution of radially increasing in green and the distribution of radially decreasing in orange

The generated synthetic trees and simulated perfusion results are shown in Fig. 4. Due to the different metabolic demand distribution, different vessel networks are produced for the two cases. For the tissue with radially decreasing metabolic demand, synthetic trees with larger radii of vessels at the center were generated. For the tissue with radially increasing metabolic demand, however, the synthetic trees exhibit smaller radii of vessels at the center and the radius values increase as one moves away from the center. For the peripheries, however, the radius values decrease again, even for the case of radially increasing metabolic demand. This can be explained by the fact that the terminal segment location is dependent on the volume mesh resolution for simplicity. As the volume mesh refines towards the surface of the tissue, the unit tissue volume perfused by each terminal segment which corresponds to the mesh element size is smaller, resulting in smaller terminal segments. The source flow and perfusion territory, however, is not affected by the mesh resolution as shown in Fig. 4. Even though the metabolic demand of the tissue is non-uniform, the flow distributions of the two source locations are the same and the generated tree structures are symmetric due to the fact that two source locations have the same radius values.

#### Comparison with a constrained constructive optimization algorithm

The tree network generated with the proposed methods is compared with the network generated with a constrained constructive optimization algorithm. Synthetic trees were generated with different tree resolutions for two algorithms: the constrained constructive optimization (CCO) and growth-based tree generation algorithm (GBO). The number of generated synthetic terminal segments varies between 567, 1189, 2369, and 5003. The metabolic demand of the tissues is uniform at 1.0 ml/min/g and the tissue density is uniform at 1.05 g/cm$^{3}$. The perfusion simulation was conducted assuming the permeability coefficient is 0.000107/mmHg/s. The sink pressure is set at 0 mmHg and the pressure-coupling coefficients $\beta _{{source}}$ and $\beta _{{sink}}$ are set to 0.000281/mmHg/s and 0.00117/mmHg/s. For the performance comparison, we used an identical computing resource with an Intel Core i7-10700K CPU 3.80 GHz with 32 GB RAM. Note that only a serial computation is compared as the CCO algorithm can be run in serial only. GBO can be executed in parallel, reducing the execution time further.

**Table 2:** Comparison of the performance and vascular tree volume for two different synthetic tree generation algorithms

Method	Number of terminal segments	567	1189	2369	5003
CCO	Execution time (min)	1.90	10.4	68.3	457.4
CCO	Total synthetic volume (ml)	22.81	25.44	27.08	29.42
GBO	Execution time (min)	0.23	0.77	3.47	17.3
GBO	Total synthetic volume (ml)	23.77	25.24	26.34	28.54

CCO stands for a constrained constructive optimization whereas GBO refers to a growth-based optimization

The execution time and total vascular volume are reported in Table 2. The execution time of the two algorithms increases as the number of terminal segments increases. The execution time of GBO is a lot faster than that of CCO. The CCO algorithm executes optimization for a global tissue domain and is in general expensive to execute. GBO executes optimization for the localized, growing tissue partitions and the computation is not as demanding as that of the constrained constructive optimization algorithm. The execution of GBO can be further accelerated as they can be executed in parallel.

The vascular volume generated with CCO and GBO is comparable with less than five percent difference. Therefore, GBO seems to be a better choice for generating synthetic trees as it generates similar tree structures as the CCO algorithm while reducing computation time significantly as demonstrated in Table 2.

**Fig. 5:**
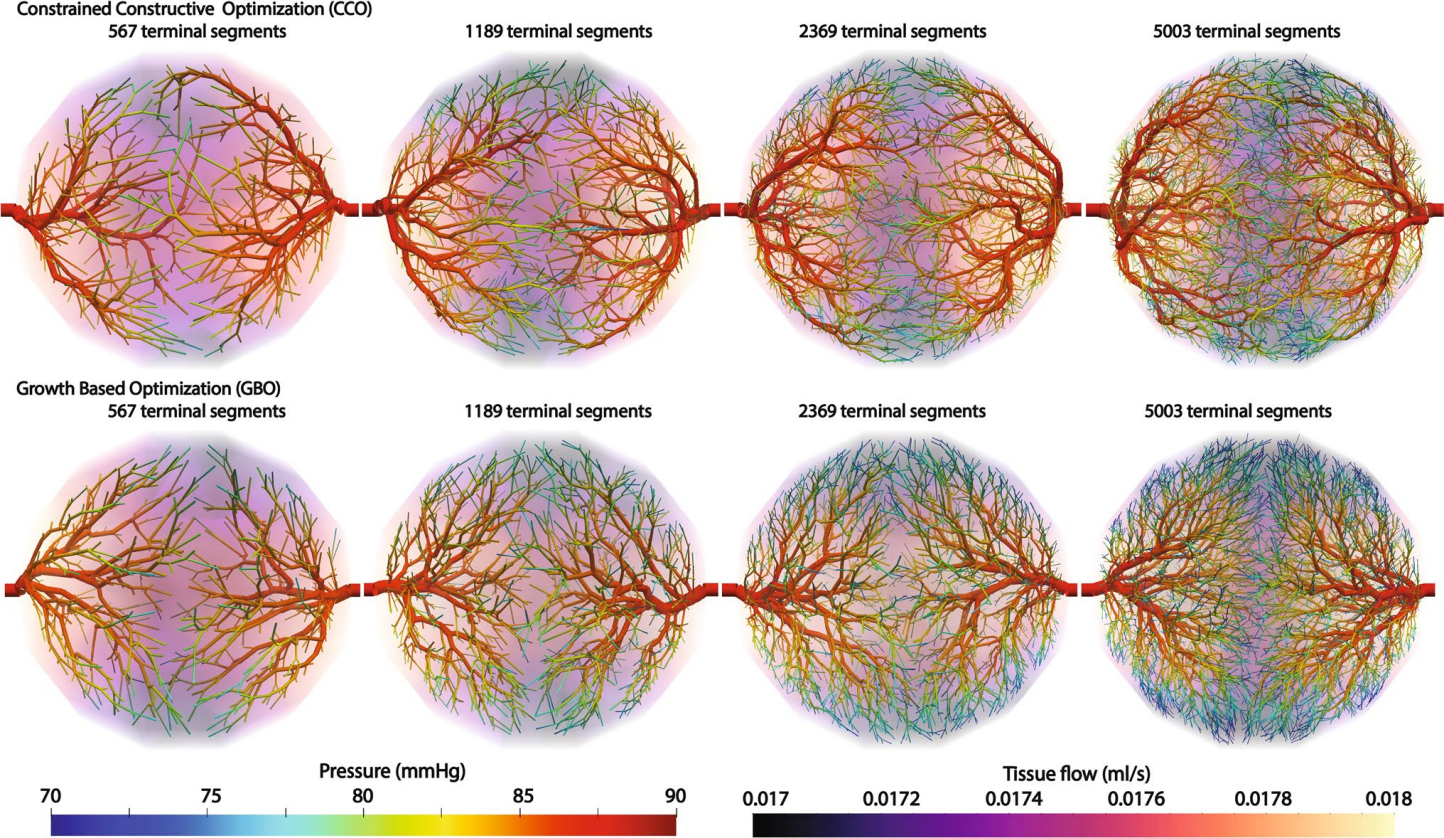
Generated synthetic tree networks and simulated perfusion with a constrained constructive optimization (top) and tissue growth-based optimization (bottom). Pressure distribution of the synthetic trees is displayed on the surface of the synthetic trees whereas the tissue flow per each volume mesh element is plotted for the tissue domain

Figure 5 compares the generated vascular trees and perfusion results for the two tree generation algorithms. One of the limitations of the CCO algorithm is the randomness of the perfusion territories. The perfusion territories are defined for each segmented coronary artery by collecting all the tissue partitions supplied by the descendants of the artery. The computed perfusion territories with different tree resolutions are slightly different for the CCO algorithm compared to the territories generated with the GBO algorithm. This difference can be more outstanding when applying this algorithm to patient-specific, complex geometries. In previous studies, when the CCO algorithm was implemented for multiple sources, the generated vascular networks sometimes showed unrealistic tree structures, supplying blood to distant tissues (Jaquet et al. 2019; Papamanolis et al. 2021). This happens as the CCO algorithm randomly selects terminal segment locations and they may therefore supply distant tissues prior to nearby tissues. With the GBO algorithm, however, the trees are generated in the nearby tissues first and grow with the tissue. Thus, the generated vessels with the GBO algorithm supply similar perfusion territories at the source locations independent of the number of terminal segments.

**Table 3:** Comparison of the performance and total vascular tree volume with different number of processors when generating 5003 terminal segments using a growth-based optimization (GBO) algorithm

Number of processors for 5003 terminal segments	1	2	3	4
Execution time with GBO (min)	17.3	10.37	7.85	6.70
Total synthetic volume with GBO (ml)	28.54	28.54	28.54	28.54

Finally, the execution time and total vascular volume with different numbers of processors are reported in Table 3. The GBO algorithm can be executed in parallel without introducing any changes in total volume of the synthetically generated vessels. The execution time decreases with an increase in the number of computing processors. The scalability is less than one as some parts of the algorithms need to be executed in serial but the tree generation can be executed in parallel, enabling the generation of patient-specific, complex, synthetic trees in a more reasonable time frame.

### Synthetic Tree Generation and Perfusion Simulation of a patient-specific cerebrovascular model

The tree generation was conducted for a patient-specific cerebrovascular model with the left and right brain hemispheres. The metabolic demand of the brain tissue is uniform at 0.5 ml/min/g and the tissue density is 1.04 g/cm$^3$ (Abe et al. 2008; Fantini et al. 2016). The total brain tissue volume is 907 ml and the assigned total baseline flow is 7.86 ml/s. The segmented cerebrovascular arteries have 58 outlets and the generated synthetic tree network has 9713 synthetic terminal segments and 19,368 synthetic segments in total. The total synthetic tree volume is 11.5 ml. The perfusion simulation was conducted assuming the permeability coefficient is 0.000107/mmHg/s (Chapelle et al. 2010; Papamanolis et al. 2021). The sink pressure is set at 0 mmHg and the pressure-coupling coefficients $\beta _{{source}}$ and $\beta _{sink}$ are uniform at 0.000269 ml/mmHg/s and 0.000553 ml/mmHg/s, respectively.

**Fig. 6:**
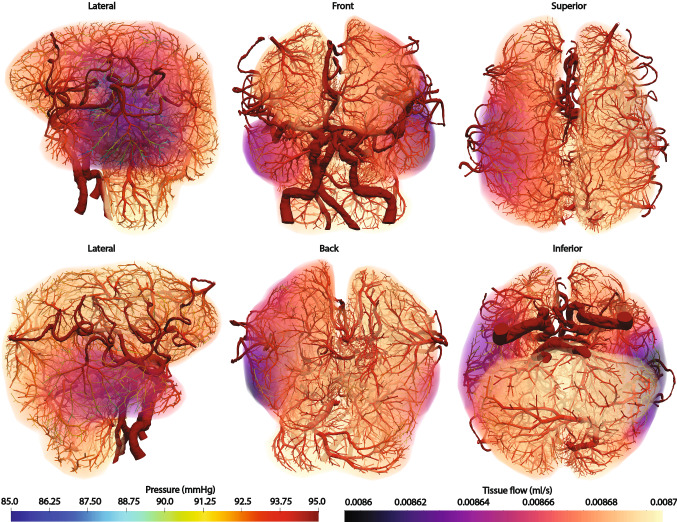
Generated synthetic tree networks and simulated perfusion on brain tissues for patient-specific cerebrovascular arteries. Pressure distribution of the synthetic trees is displayed on the surface of the synthetic trees whereas the tissue flow per each volume mesh element is plotted for the tissue domain. Note the tissue flow varies little between 0.0086 and 0.0087 ml/s

Figure 6 shows the synthetic trees generated for the cerebrovascular arteries with a volume rendering of perfused tissue flows. As the segmented cerebrovascular arteries are healthy, the perfused tissue flow is uniform with little variation. The tree density is not uniform as the terminal segment locations of the terminal segments were assigned to the centroids of the tissue volume mesh for simplicity. Even though the synthetic tree density is dependent on the mesh resolution, the terminal segment diameter is dependent on the metabolic demand. To comply with the uniform metabolic demand, the diameter of vessels and terminal segments is reduced at locations of dense terminal locations.

### Synthetic Tree Generation and Perfusion Simulation of a patient-specific coronary model

**Fig. 7:**
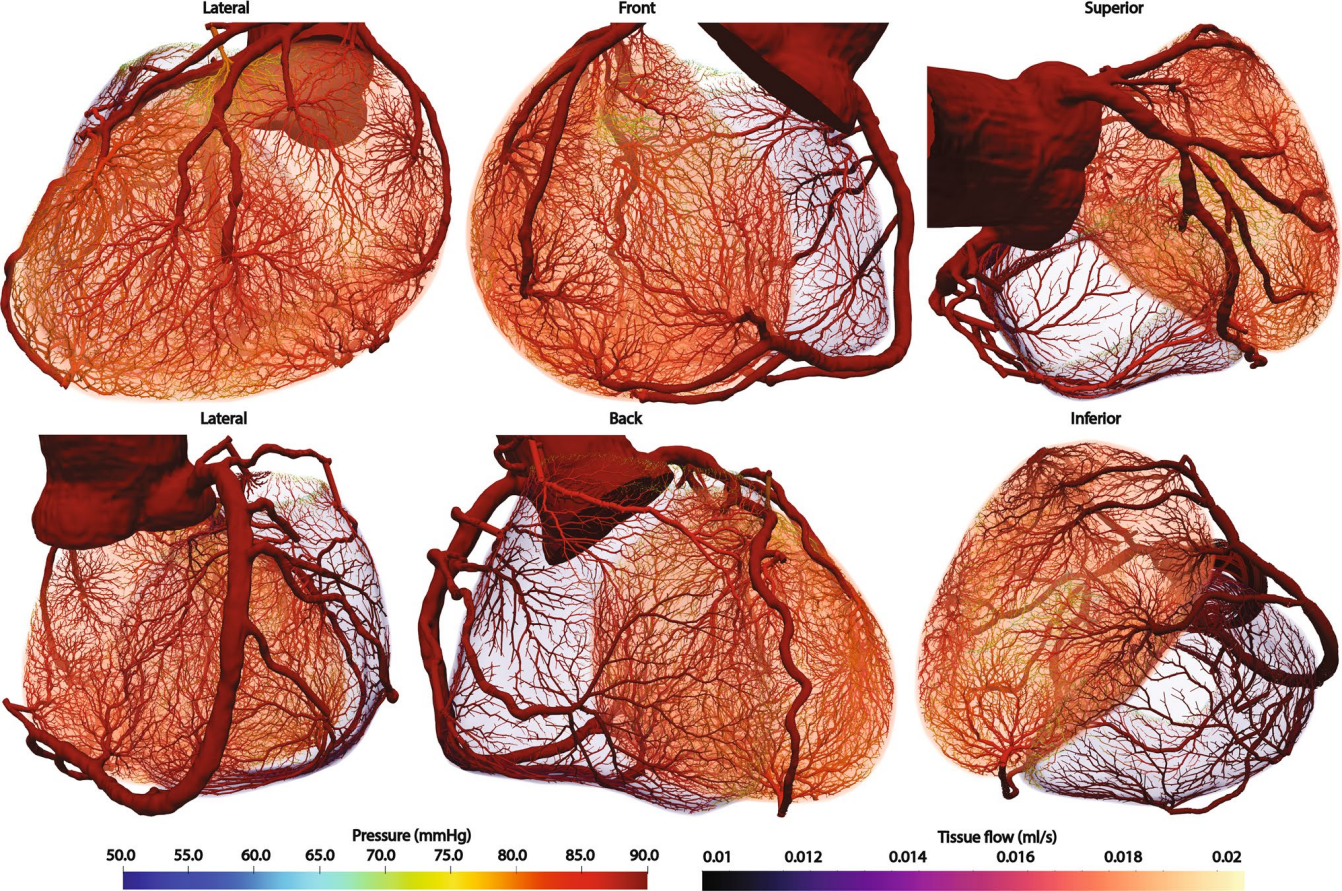
Generated synthetic tree networks and simulated perfusion on the left and right ventricles for patient-specific coronary arteries. Pressure distribution of the synthetic trees is displayed on the surface of the synthetic trees whereas the tissue flow per each volume mesh element is plotted for the tissue domain

The tissue-growth based tree generation methods were applied to a patient-specific coronary artery model with the left and right ventricle tissue models. The metabolic demand of the left ventricle is uniform at 1.0 ml/min/g and that of the right ventricle is uniform at 0.65 ml/min/g (Bakkum et al. 2015; Liu et al. 2018). The segmented coronary arteries have 26 outlets in total, 18 of them exclusively supply blood to the left ventricle while the remaining 8 outlets supply blood to the right ventricle. Those outlets are assumed to supply to one tissue only for simplicity. The outlets supplying blood to the right ventricle supply 0.37 ml/s in total whereas the outlets supplying blood to the left ventricle supply 2.96 ml/s in total. The total perfusion volume of the right ventricle is 32.7 ml and that of the left ventricle is 169.3 ml. The generated synthetic trees contain 17,633 terminal segments for the left ventricle and 10,811 for the right ventricle. The total number of generated synthetic tree segments is 35,321 for the left ventricle and 21,661 for the right ventricle. The synthetic tree volume feeding the left ventricle is 11.8 ml whereas the synthetic tree volume feeding the right ventricle is 0.7 ml. The perfusion simulation was conducted assuming the permeability coefficient is 0.000107/mmHg/s (Chapelle et al. 2010; Papamanolis et al. 2021). The sink pressure is uniform at 0 mmHg and the pressure-coupling coefficients $\beta _{source}$ and $\beta _{sink}$ for the left ventricular tissue are set at 0.000269/mmHg/s and 0.00120/mmHg/s. The pressure-coupling coefficients $\beta _{source}$ and $\beta _{sink}$ for the right ventricular tissue are set at 0.000281/mmHg/s and 0.000747/mmHg/s. Figure 7 demonstrates that the synthetic trees have denser tree resolution and relatively bigger vessels for the left ventricle compared against the trees of the right ventricle as the left ventricle has higher metabolic demand and a bigger volume.

**Fig. 8:**
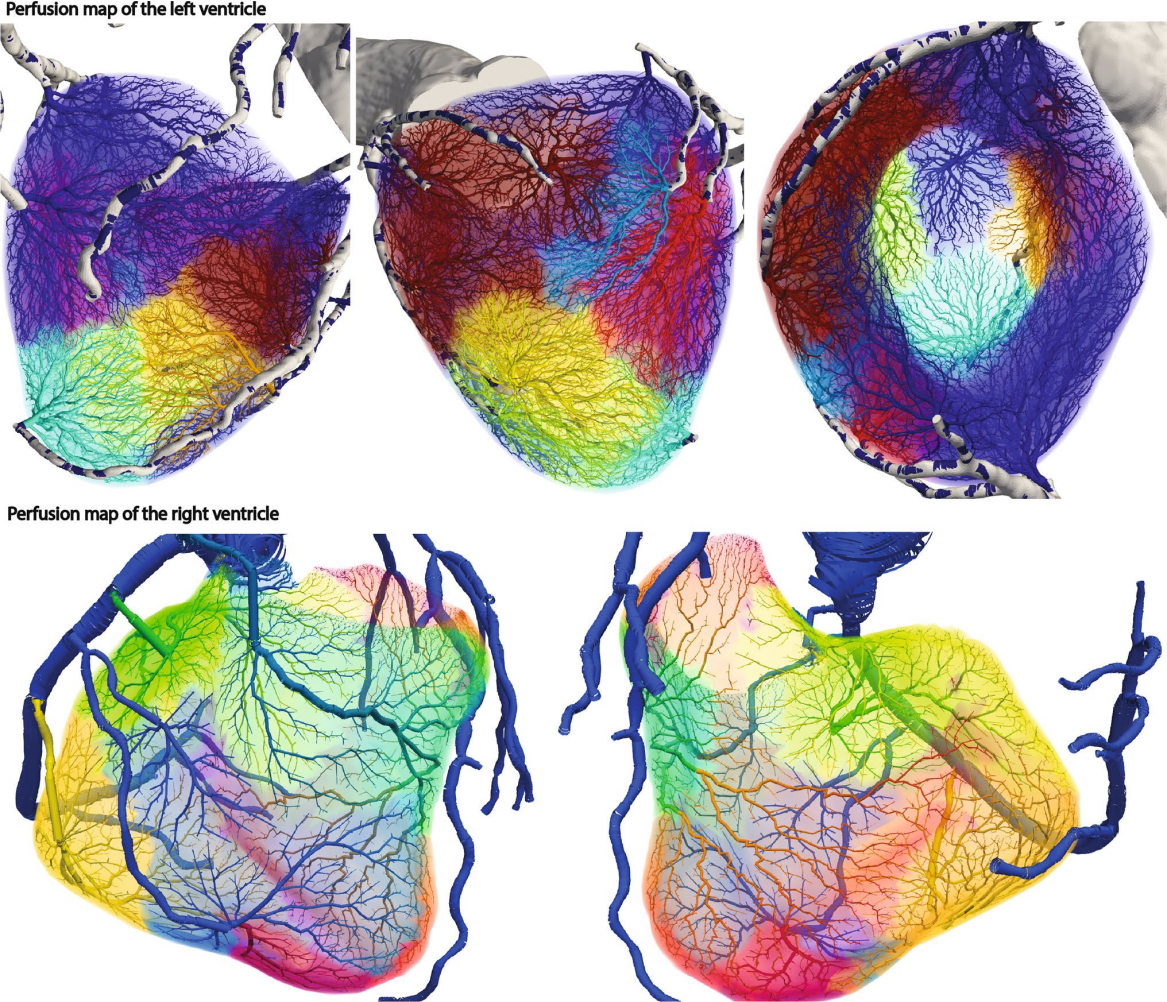
Perfusion territories of the left and right ventricles supplied by the segmented coronary arteries. The color of each perfusion territory is associated with the coronary artery which supplies blood to it

Figure 8 shows the perfusion territories supplied by each segmented coronary artery. Using the tissue-growth based tree generation algorithms, the synthetic vessels are generated for nearby tissues first. Thus, arteries perfuse nearby tissues first. As demonstrated in various experimental studies, the association between the blood vessels and the perfusion territories is highly dependent on the distance between the two Ide et al. (2017), Kang et al. (2019), Kim et al. (2016), Kurata et al. (2015), Malkasian et al. (2018), van den Wijngaard et al. (2013) and Van Horssen et al. (2016). The generated perfusion color map demonstrates realistic perfusion territories as they map major vessels to nearby tissues. As the tree generation algorithms rely on the metabolic demand of the perfusing tissue and the competition with neighboring vessels, the perfusion territories have various volumes and shapes.

## Discussion

We have demonstrated a synthetic tree generation algorithm based on physiological principles whereby the tissue growth and increased metabolic demand trigger the angiogenesis process. The generated synthetic trees supply blood to nearby tissues similar to the findings observed in actual anatomies. Additionally, as the tree growth depends on the diameter of the source locations, the generated synthetic vessels obey the allometric scaling laws (West et al. 1997). The tissue-growth based tree generation algorithm was successfully executed on idealized geometries with a uniform metabolic demand for different tree resolutions. The two source locations supplied the same amount of flow to the tissues for different tree resolutions as the diameter of the two source locations is the same. The perfusion territories at the source locations were also independent of the number of generated terminal segments. Next, the tree generation algorithm was run on the idealized tissue with non-uniform metabolic demands. When there was increased metabolic demand as the tissue approaches the center or vice versa, the generated synthetic trees were adapted to supply blood to the tissues based on their metabolic demands. For the tissue with radially decreasing metabolic demand, the synthetic tree generation algorithm developed bigger tree structures toward the center of the tissues. The vessels supplying the periphery of the tissues were relatively atrophied as the metabolic demand of the peripheral tissue was diminished. For the case of radially increasing metabolic demand, opposite tree structures were observed. These idealized cases demonstrated the capability of the tissue-growth based synthetic tree generation to construct trees while interacting with the tissues which they supply blood to. The algorithm is capable of adapting tree structures depending on the growth and metabolic demand of the tissue.

Further, the tree generation algorithm was applied to two actual patient-specific geometries. First, an application to a cerebrovascular system was performed to demonstrate the capability to construct synthetic trees on a complex geometry obtained from medical image data of a brain. The synthetic trees on the left and right brain hemispheres demonstrated that the trees can be constructed based on the growth and the metabolic demand of the brain tissues. For this study, we applied a uniform metabolic demand for the generation of the synthetic trees and conducted the perfusion simulation. The results demonstrated uniform tissue perfusion with small variations as expected from a healthy subject. As another real, patient-specific application, the coronary artery system was chosen. The synthetic trees for the left and right ventricles developed different tree structures as they have different metabolic demands. The synthetic trees developed smaller diameters and less dense networks on the right ventricle due to its lower metabolic demand.

The execution time of the tissue growth-based tree generation algorithm is reduced up to 26 folds compared to that of the constraint constructive optimization algorithm. We performed the comparison study between the growth-based optimization (GBO) and constrained constructive optimization (CCO) algorithms for the generation of 567, 1189, 2369, and 5003 terminal segments and the execution time reduced by 8, 13, 20, and 26 folds, respectively. The execution time is further reduced as the number of segments increases. Both the GBO and CCO algorithms require iterative search for an optimal tree configuration within a prescribed search distance and the number of candidate tree configurations grows exponentially as the number of segments increases. When applying the GBO algorithm, however, we can effectively reduce the number of computed tree configurations to the subtrees in the vicinity of actively growing tissue partitions and significantly reduce the execution time. And as the GBO algorithm can be executed in parallel, the execution time can be further reduced. This optimized algorithm is successful in finding a minimum total vascular volume as well. The vascular volume generated using the CCO algorithm is either comparable or slightly higher compared to the volume generated using the GBO algorithm, exhibiting differences of less than five percent for the idealized geometries. The reason why the total vascular volume using a CCO algorithm has a tendency to be larger than the volume using the GBO algorithm seems to be due to the search constraints. When the CCO algorithm seeks for a global minimal volume it constrains search bounds from a big domain initially and scales back to a small domain to reduce the time spent in the search. Additionally, there is randomness when setting terminal segment locations for initial iterations which influences search domains of the subsequent iterations. Depending on how this search length is designed, the optimization algorithm determines the subspace where a local minimum of the loss function is sought. Jessen et al. also reported that the CCO algorithm searches for a locally optimal tree configuration and obtained a more optimal tree structure by expanding the search space using the nonlinear programming based optimization methods (Jessen et al. 2022). For the GBO algorithm, however, there is less randomness in the algorithm as it relies on the tissue growth. Depending on how the growth rate of the tissue is set, the generated vessels will have different structures. Further, as the algorithm minimizes the loss function for each iteration of the tissue growth, synthetic trees are constructed in an optimal fashion when supplying blood to the nearby tissues.

When the GBO algorithm seeks for a minimal volume for partially grown tissues—as the tissue grows from a small volume supplied by the initial source location to the final tissue—it can be projected to be computationally demanding as the tissue dynamically grows and optimal vascular networks are sought for each iteration. The computation, however, is a lot cheaper compared to that of a CCO algorithm as it is constrained to actively growing tissue partitions only. Furthermore, the computation can be optimized by parallelization. In comparison to existing tree generation approaches, the GBO algorithm generates synthetic trees more quickly while utilizing the physiological principles of the custom and nonlinear programming based CCO algorithms. The CCO algorithms sometimes fail to represent anatomically realistic vessels by generating trees to rather distant tissues. With the GBO algorithm on the other hand, the tissue and vessel growth happens in tandem and the vessels supply nearby tissues first. Compared to the staged growth-based CCO algorithms, the GBO algorithm is robust as the tissue and vessels grow interactively. Furthermore, the GBO algorithm satisfies the minimum work principle in addition to the supply–demand relationship which is not necessarily satisfied by the supply–demand relationship-based tree generation methods. Lastly, the GBO algorithm is versatile to model trees in different organs and with different morphological characteristics by adjusting the metabolic demand and the tissue growth rate as well as the direction of the tissue.

There are limitations of this study that must be addressed in future work. First, the proposed tree generation methods rely on the assumption that the segmented vessels are the original source locations where the tissue starts growing from. We start from the source location with a prescribed small vessel diameter and a unit tissue which is supplied by this source and start growing the tissues and the vessels. If the initial source location at the beginning of the tissue growth is different, the generated tree structure can change significantly. Second, the location of the generated terminal tree segments is dependent on the discretized tissue volume mesh for the results presented in this study. We assumed that each centroid of the finite element mesh of the tissue domain is a candidate location for a new terminal segment. Therefore the tree structure highly depends on the mesh quality of the tissue volume mesh. Due to this simplification, anisotropic tissue volume meshes result in non-uniformly generated tree structures. The tissue volumes of the patient-specific models have uneven mesh resolution for certain regions and have contributed to the generation of uneven synthetic trees. However, as the generated synthetic vessel diameters are set according to the metabolic demand of the tissue, the tree vascular volume is not much affected by this uneven mesh density. However, we plan to address this issue in the future. Third, the synthetic tree generation relies on the segmented arteries as they serve as the source locations. Vessels with a size below the image resolution are overlooked and are not modeled. In the applications treated in this paper, there are perforating arteries present in both the coronary and the cerebrovascular systems. We did not model these perforating arteries as additional source locations. As adding these perforating arteries can introduce changes in the perfusion and blood flow distribution, future work will address these unseen vessels before starting the synthetic tree generation. Fourth, the perfusion model utilized in this study is a single compartment Darcy flow model. Depending on the resolution of the synthetically generated vessels the perfusion model can contain vessels of different scales. As the flow and material characteristics of these vessels are heterogeneous, they need to be modeled with different compartments. Many studies have reported limitations of a one-compartment Darcy flow model due to the dependency on the mesh resolution and failure to model scale-separation, to list a few (Hyde et al. 2014; Michler et al. 2013; Lee et al. 2015). Future work will address these limitations by implementing a multi-compartment Darcy flow model. Lastly, more research and validation work will be conducted by applying the proposed methods to patient-specific geometries and validate against measurement data. Patient-specific validation, however, is limited by the resolution of available in-vivo image techniques which allows to validate the morphology of arteries and larger arterioles only. For major vessel networks like coronary and cerebrovascular beds, morphology studies that use ex-vivo data acquired from humans or animals are available for validation. They are however not specific to the patient.

## Conclusion

We developed a tissue-growth based synthetic tree generation algorithm and a perfusion simulation framework for the generation of vascular networks below the medical image resolution and perfusion simulation using the interactions between the blood vessels and the supplied tissues. The presented methods can generate realistic vascular networks as a function of metabolic demand of perfused tissues. Further, the algorithm is a lot faster compared to the execution time of a constrained constructive optimization algorithm and can be parallelized to expedite the execution. We successfully simulated perfusion of tissues by coupling the generated vessel networks to a Darcy flow model which approximates the small arteriole and capillary blood flow using a porous medium. The methods were demonstrated with ideal geometries with uniform and non-uniform metabolic demands and two patient-specific geometries—cerebrovascular arteries with segmented brain tissues and coronary arteries with left and right ventricles—to demonstrate that realistic synthetic trees can be generated promptly by considering local metabolic demand of perfused tissues.
